# Electrochemical Switching of Metallopolymer-Functionalized Indium Tin Oxide Derived by Cu^**0**^‑Mediated Atom Transfer Radical Polymerization

**DOI:** 10.1021/acsapm.5c02861

**Published:** 2025-09-09

**Authors:** Jaeshin Kim, Bizan N. Balzer, Markus Gallei, Suteera Witayakran

**Affiliations:** † Polymer Chemistry, 9379 Saarland University, Campus C4 2, Saarbrücken 66123, Germany; ‡ Institute of Physical Chemistry, University of Freiburg, Albertstr. 21, Freiburg 79104, Germany; § Freiburg Materials Research Center (FMF), University of Freiburg, Stefan-Meier-Str. 21, Freiburg 79104, Germany; ∥ Saarene, Saarland Center for Energy Materials and Sustainability, Saarland University, Saarbrücken 66123, Germany; ⊥ Max Planck Institute for Informatics, Saarland Informatics Campus, Building E1 4, Saarbrücken 66123, Germany

**Keywords:** polymer brushes, ferrocene, redox-responsive polymers, conductive surfaces, polymer immobilization

## Abstract

A redox-responsive polymer-modified indium tin oxide (ITO) was fabricated via a convenient filter paper-assisted Cu^0^-mediated surface-initiated atom transfer radical polymerization (FP-Cu^0^-SI-ATRP), enabling the formation of ferrocene-containing polymer brushes, poly­(2-(methacryloyloxy)­ethyl ferrocenecarboxylate) (PFcMA), with tunable thickness on ITO. By varying the monomer concentration, uniform polymer brushes with controllable thicknesses ranging from 10 to 122 nm were obtained. Compared to surface-initiated atom transfer radical polymerization (SI-ATRP), FP-Cu^0^-SI-ATRP achieved significantly higher polymerization rates, thicker films, and a shorter reaction time (5 h vs 20 h), while eliminating the need for subsequent metal catalyst removal. Characterization by Fourier Transform Infrared spectroscopy (FTIR), Ultraviolet–visible spectroscopy (UV–vis), atomic force microscopy (AFM), an ellipsometer, and water contact angle (WCA) measurements confirmed the successful grafting and systematic changes in PFcMA brush thickness and surface properties. Electrochemical performance, assessed by cyclic voltammetry (CV), revealed that thinner films exhibited efficient, diffusion-controlled redox behavior, whereas thicker films showed increased resistive effects. While the modified ITO prepared via SI-ATRP displayed lower redox activity despite having a similar thickness, this suggests a less favorable polymer brush morphology for charge transport. These findings establish FP-Cu^0^-SI-ATRP as a promising approach for constructing redox-active interfaces with tunable electrochemical properties for smart material applications.

## Graphical abstract

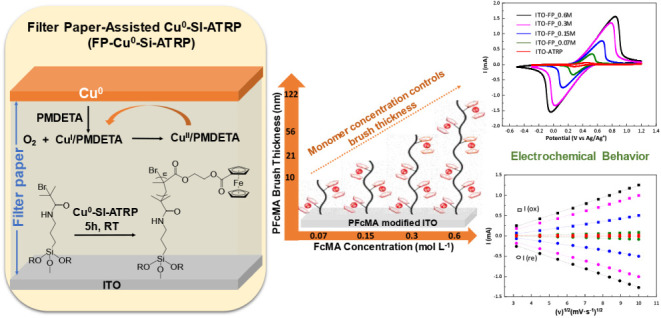

## Introduction

Polymer brushes—assemblies of polymer chains tethered by one end to a substrate—have become a cornerstone in the design of functional surfaces due to their tunable chemical, physical, and mechanical properties.^1^ Among various substrates, indium tin oxide (ITO) has attracted considerable interest. ITO is a prominent transparent conducting oxide (TCO) that combines high optical transparency in the visible range (>80%) with low electrical resistivity (∼10^– 4^ Ω·cm), making it indispensable for applications in modern optoelectronics.^2,3^ Composed of a mixture of indium oxide (In_2_O_3_) and tin oxide (SnO_2_), ITO exhibits a wide bandgap (3.5–4.3 eV), which allows for visible light transmission while maintaining good electrical conductivity. This combination makes ITO a suitable material for flat panel displays, solar cells, light-emitting diodes (LEDs), smart windows, and biosensors.^4–8^ However, despite its advantageous properties, the native surface of ITO often suffers from issues such as poor chemical stability, limited functionalization capability, variable work function, and poor compatibility with organic or polymeric materials.^8–10^ These drawbacks have stimulated extensive research into surface modification techniques, including chemical treatments (e.g., acid/base washes),^11^ physical techniques (e.g., plasma or UV-ozone treatments),^11,12^ and the deposition of thin organic or polymeric interlayers,^13–18^ aimed at enhancing ITO’s interfacial compatibility, stability, and functionality in advanced devices.

One particularly promising approach is the modification of ITO with polymers, which provides a high degree of flexibility in tuning surface properties such as wettability, surface energy, electronic alignment, and chemical functionality. Polymers can be applied via physisorption, covalent bonding through functional silanes or phosphonic acids,^19^ or more advanced “grafting from” techniques^1,20^ such as surface-initiated atom transfer radical polymerization (SI-ATRP),^21–24^ living anionic polymerization techniques,^25–28^ or reversible addition–fragmentation chain-transfer (RAFT) polymerization.^29–31^ Grafting polymer brushes onto ITO surfaces provides a powerful platform for integrating the electrochemical properties of ITO with the responsive functionalities of polymers. Surface-initiated polymerization techniques, particularly SI-ATRP, have enabled precise control over brush architecture, thickness, and composition. These controlled techniques enable the creation of stimuli-responsive, biocompatible, and electroactive interfaces, leading to the development of materials for various advanced applications.^1,32^ For example, hole-transporting polymer brushes, such as poly­(*N*-vinylcarbazole) (PVK), were grafted onto ITO electrodes to improve charge transport in organic electronic devices.^33^ Polymer brushes incorporating phenothiazine units were grafted onto ITO via SI-ATRP. These redox-active brushes exhibited reversible electrochemical behavior and enhanced nanoscale mechanical stability, indicating their potential for applications in energy storage and sensing technologies.^34^ In addition, conductive polythiophene-based brushes on ITO substrates can be fabricated using a self-templating approach. These brushes exhibited high conductivity after chemical doping, attributed to their ordered structure and extended conjugation length. Such properties make them promising candidates for optoelectronic applications where directional conductivity is essential.^35^

Moreover, the integration of redox-active monomers, such as ferrocene derivatives, into polymer brushes on a substrate surface has garnered significant interest, leading to systems with switchable surface properties attributed to their reversible redox activity (Fe^2+^/Fe^3+^) when exposed to an external voltage or redox environment. These brushes can reversibly change their hydrophilicity, permeability, and molecular binding affinity, enabling advanced functionalities in controlled drug delivery, bioelectronic interfaces, smart surfaces, biosensors, and electrochemical sensing.^36,37^ To date, a variety of ferrocene-containing polymer brushes have been successfully grafted onto ITO through SI-ATRP. These include poly­(ferrocene ionic liquid),^38^ poly­(ferrocenyl quaternary ammonium),^39^ polyferrocenylmethyl methacrylate (PFMMA),^40–43^ polyferrocenylbutyl methacrylate (PBMA),^41,43^ polyferrocenylnonyl methacrylate (PFNMA),^41,43^ and poly­(2-(methacryloyloxy)­ethyl ferrocenecarboxylate)­(PFcMA).^44^

More recently, copper^0^-mediated surface-initiated atom transfer radical polymerization (Cu^0^-SI-ATRP) has been introduced as a variant of the classical ATRP method.^45,46^ Unlike conventional Cu­(I)-based ATRP systems, which often require stringent deoxygenation and relatively high catalyst loadings, Cu^0^-SI-ATRP operates efficiently in open-air conditions, at ambient temperature, and with minimal copper content, thanks to the continuous regeneration of the active Cu­(I) species from the copper metal surface. Its compatibility with a broad range of monomers, including acrylates, methacrylates, and acrylamides, enables the synthesis of functional coatings tailored for specific applications.^47–50^ These characteristics have made Cu^0^-SI-ATRP particularly attractive for surface modifications where environmental control is challenging, as well as for applications where minimizing catalyst residue is essential. In surface-initiated polymerization, Cu^0^-SI-ATRP has been widely employed to generate polymer brushes from planar substrates, such as silicon (Si) wafers,^51–56^ glass,^54^ polymer membranes,^57^ and hydrogels.^58^

To broaden the scope of this polymerization approach, we suggest exploring Cu^0^-SI-ATRP on other substrates, including ITO. In this work, ferrocene-containing polymer brushes (PFcMA) were grafted from ITO surfaces via Cu^0^-SI-ATRP to combine ITO’s electrochemical properties with the redox-responsive features of the polymer. To our knowledge, this is the first study to report the grafting of ferrocene-containing polymer brushes onto ITO using Cu^0^-SI-ATRP. We evaluated three different methods—load-, spacer-, and filter paper-based approaches—to determine the most suitable polymerization technique for grafting PFcMA brushes onto planar substrates, using Si wafers as model substrates for direct comparison. Next, the chosen method, the filter paper-assisted Cu^0^-SI-ATRP, which allowed precise control over the PFcMA brush thickness and provided faster polymerization, producing the thickest brushes in the same reaction time, was further utilized to modify ITO surfaces. Since this method allows precise tuning of the polymer brush thickness, thereby enabling systematic investigation of thickness-dependent electrochemical properties. Cyclic voltammetry (CV) was employed to characterize and compare the redox behavior of ITO modified with PFcMA brushes of varying thicknesses. For comparison, a PFcMA brush-modified ITO with a similar thickness was also prepared from SI-ATRP. The study aims to elucidate how the structural characteristics of the polymer film, particularly its thickness, and polymerization methods influence the redox switching performance and electron transfer kinetics, offering valuable insights for the design of advanced smart electrochemical interfaces.

## Experimental Section

### Materials

All chemicals were used as received unless otherwise stated. 3-Aminopropyl-triethoxysilane (APTES), dimethyl sulfoxide (DMSO), and tetrahydrofuran (THF) were purchased from Fluka. Triton X-405 solution, copper­(I) chloride (CuCl), *N,N,N′,N′,N″*-pentamethyldiethylenetriamine (PMDETA), α-bromoisobutyryl bromide (BIBB), *tert*-butyl 2-bromoisobutyrate (*t*BbiB), tetrabutylammonium hexafluorophosphate ([TBA]­[PF_6_]), and triethylamine (TEA) were purchased from Sigma-Aldrich. Dichloromethane (DCM), sulfuric acid (H_2_SO_4_), and aluminum oxide (Al_2_O_3_, neutral) were purchased from Fisher Scientific. Methanol (MeOH), ethanol (EtOH), acetonitrile (ACN), and hydrogen peroxide (H_2_O_2_, 33 wt %) were purchased from VWR Chemicals. Anisole was purchased from Carl Roth. DCM and anisole were dried over 3 Å molecular sieve before use. For the SI-ATRP, CuCl was washed five times with glacial acetic acid and ethanol. PMDETA, anisole, and *t*BbiB were degassed and stored in a glovebox. The copper complexes and *t*BbiB solution were freshly prepared in anisole and treated in the glovebox. 2-(Methacryloyloxy)­ethyl ferrocenecarboxylate monomer (FcMA) was synthesized as reported elsewhere.^44^ Si wafers (single-side polished, ⟨100⟩, N-type, diameter = 125 mm, thickness: 590–660 μm) were purchased from Siltron. ITO glass substrates with a sheet resistance of 14–16 Ω·cm^–2^ were purchased from Ossilla, UK.

### Substrate Cleaning

For Si wafers, the substrates, consisting of two pieces measuring 1 × 1 cm^2^ and one piece measuring 1 × 2 cm^2^, were first sonicated in ethanol and Milli-Q water (ultrapure water, 18.2 MΩ·cm at 25 °C) for 5 min each. They were then treated with Piranha solution (H_2_SO_4_:H_2_O_2_ = 3:1) *(Warning: Piranha solution is highly corrosive and should be prepared and used with extreme caution. Never store the solution.)* at 150 °C for 30 min and extensively washed with Milli-Q water and EtOH before being dried under a stream of nitrogen (N_2_). The obtained substrates were further cleaned by exposure to oxygen plasma (Diener Electronic, Femto SLS) for 3 min to remove any organic substances from the surface.

For ITO, the substrates, consisting of two pieces measuring 0.75 × 1 cm^2^, one piece measuring 1.5 × 2 cm^2^, and one piece measuring 2.5 × 2.5 cm^2^, were initially cleaned through sonication in successive solutions of Triton X-405 in water, Milli-Q water, and EtOH for 15 min each, followed by drying under N_2_ stream. The substrates were then treated with oxygen plasma (Diener Electronic, Femto SLS) for 15 min to eliminate any remaining organic contaminants.

### Deposition of APTES on the Substrate Surface

The plasma-treated substrates were then subjected to the APTES deposition step. The aqueous deposition method^59^ was used in this study (Figure 1a). Briefly, the APTES stock solution composed of 50% MeOH, 47.5% APTES, and 2.5% Milli-Q water was prepared and kept at 4 °C for at least 1 h before use. The stock solution was then diluted 1:500 in MeOH. Subsequently, the plasma-treated substrates were immersed in the diluted stock solution (15 mL for Si wafers and 30 mL for ITO) at room temperature for 30 min, and then rinsed with MeOH, dried under a flow of N_2_, and heated in the oven at 110 °C for 30 min.

**Figure 1:**
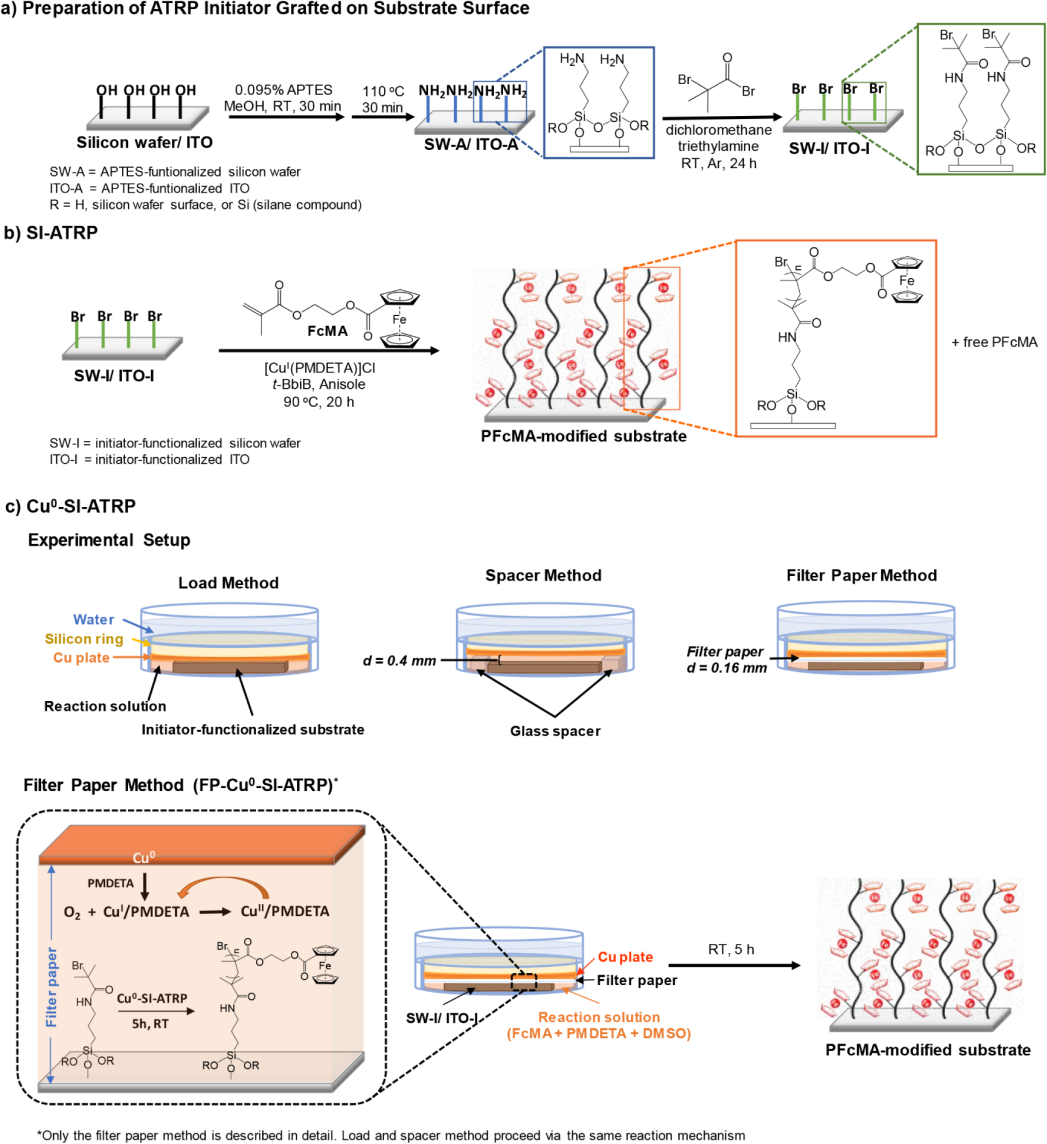
Schematic illustration of a) APTES deposition followed by ATRP initiator functionalization on substrate surface, b) PFcMA polymer brush fabrication via SI-ATRP, and c) PFcMA polymer brush fabrication via Cu^0^-SI-ATRP; top: experimental setup for three different approaches, bottom: the reaction of filter paper method as representative (load and spacer method follow an identical mechanism and are therefore not shown).

### Preparation of ATRP Initiator Grafted on Substrate Surface

The APTES-functionalized substrates (SW-A/ITO-A, Figure 1a) were carefully placed into a flame-dried Schlenk tube, which was then backfilled with argon (Ar). In a separate dried round-bottom flask, a mixture of BIBB (4 mmol) and TEA (4 mmol) in 20 mL of dry DCM was prepared and cooled to 0 °C using an ice bath. Next, the solution was degassed by sparging N_2_ for 30 min, after which it was transferred to the Schlenk flask containing the APTES-treated substrates using a syringe. The reaction was maintained at room temperature for 24 h within an inert atmosphere. Finally, the substrates were removed from the reaction flask, thoroughly washed with DCM and EtOH, and dried under a N_2_ stream.

### PFcMA Grafting from the Substrates Via SI-ATRP

SI-ATRP was carried out following the previously described method, with some modifications (Figure 1b).^60,61^ The initiator-functionalized substrates (SW-I/ITO-I) were placed in a Schlenk tube and dried under vacuum, and the flask was then refilled with Ar. In a separate Schlenk flask, anisole (10.98 mL), FcMA (2.4 g, 7 mmol), and *t*BbiB (54 mM, 0.43 mL, 0.023 mmol) were added. The mixture was then degassed by three freeze–pump–thaw cycles. Subsequently, the mixture was transferred to the argon-purged Schlenk tube containing the initiator-functionalized substrates using a syringe. The solution was heated to 90 °C for 10 min. Then, the polymerization was initiated by adding a solution of [Cu^I^(PMDETA)­Cl] (0.2 M, 0.58 mL, 0.117 mmol in anisole). The polymerization reaction proceeded at 90 °C for 20 h. After that, the PFcMA-functionalized substrates were removed and washed extensively by sonication in THF and ethanol for 5 min each, and then dried under a nitrogen flow. The reaction solution was passed through the Al_2_O_3_ column to remove the remaining copper catalyst, and the free polymer (PFcMA) was collected by precipitation with hexane and characterized by using size exclusion chromatography (SEC).

### Poly­(2-(methacryloyloxy)­ethyl ferrocenecarboxylate) (PFcMA) Grafting from the Substrates Via Cu^0^-SI-ATRP

The Cu^0^-SI-ATRP (Figure 1c) was performed using three different approaches: load-, spacer-, and filter paper-based method.^57^ The experimental setup of these methods were modified for the FcMA monomer, as it tends to recrystallize easily when the solvent evaporates slowly during the reaction. The setup for each method was illustrated in Figure 1c. Briefly, the initiator-functionalized substrates (SW-I/ITO-I) were positioned in a Petri dish with the initiator-modified side facing upward. A copper plate (99.9%, 0.3 mm thick, SOFIALXC) was first rinsed in acetic acid and subsequently washed with Milli-Q water, EtOH, and acetone, before being dried under a N_2_ stream. The reaction solutions containing FcMA monomer (0.6 M or desired concentration) and PMDETA as a ligand (0.012 M) in DMSO were prepared. In the load method, the reaction solution was gradually added to the Petri dish containing the substrates until it fully covered the substrate surface. Subsequently, a copper plate was positioned on top. For the spacer method, small pieces of glass slides served as spacers, with the substrates placed between them. The reaction solution was then carefully added to the Petri dish, covering the spacers, followed by the placement of the copper plate on top. The separation distance (d) between the substrates and the copper plate was 0.4 mm. In the filter paper method, filter paper (Fisherbrand grade 601, cellulose, 0.16 mm thick with a pore size of 5 to 13 μm) with a thickness of 0.16 mm was utilized. This filter paper was set on top of the substrates, and the reaction solution was gradually added to the Petri dish until it covered the filter paper. The copper plate was then placed over it, with the distance between the substrate surface and the copper plate equal to the thickness of the filter paper. In all these cases, a silicon ring was laid on top of the copper plate. The Petri dish was covered with a Petri dish cover, and another Petri dish filled with water was placed on top to minimize solvent evaporation during polymerization. The substrates were allowed to sit for 5 h under ambient conditions. Afterward, they were removed and cleansed via sonication in THF and ethanol for 5 min each, followed by drying under a nitrogen flow.

### Material Characterization

Fourier transformed infrared (FTIR) spectra were recorded on an attenuated total reflection Fourier transform infrared (ATR-FTIR, diamond ATR) instrument (Bruker Alpha II) in the 400–4000 cm^–1^ range with a resolution of 4 cm^–1^.

Static WCA measurements utilized 10 μL deionized water droplets, dispensed via a 100 μL Hamilton syringe on a KD Scientific syringe pump, with the aid of a custom-made xyz positioning table. Photographs were taken using a Nikon D5400 and digiCamControl 2.1.2.0, and the evaluation was performed using OpenDrop 3.3.1.^62^ The WCA was measured from five different spots on the sample. Data are presented as mean ± standard deviation (SD).

SEC was performed by utilizing a 1260 Infinity II (Agilent Technologies) and two eluents. THF was used as the mobile phase (flow rate 1 mL min^–1^), and a PSS SECurity^2^ RI/UV detector on an SDV column from polymer standard service (PSS) (SDV 10^3^ Å, SDV 10^5^ Å, SDV 10^6^ Å, 5 μm) was used. Polystyrene (PS) standards were used for calibration, and PSS WinGPC UniChrom V 8.31 was used for evaluation.

CV was performed using a BioLogic SP-150 potentiostat in a custom-built electrochemical cell. This cell was equipped with an Ag/Ag^+^ reference electrode, a Pt-wire counter electrode, and a modified ITO substrate as the working electrode. The measurements were conducted in a 0.1 M solution of tetrabutylammonium hexafluorophosphate ([TBA]­[PF_6_]) in acetonitrile, with a scan rate of 100 mV·s^–1^ over a range of −0.3 to 1.2 V. The evaluation was carried out using EC-Lab V11.46.

The Thermo Scientific Evolution 220 spectrometer was used to perform UV–vis spectroscopy of PFcMA polymer brushes on an ITO substrate. An unmodified ITO substrate served as the background. Scanning was performed over the wavelength range of 800 to 250 nm, with a data interval of 1.00, a scan speed of 266.75 nm min^–1^, and a cycle number of 1.

The dry thickness of the PFcMA polymer brushes on Si wafers was measured using a SE400adv ellipsometer (Sentech Instruments) equipped with HeNe laser light source. Data were acquired in reflection mode at angles of incidence of 70° at a fixed wavelength of 632.8 nm. All measurements were conducted at room temperature in ambient air. The thickness average was calculated from five different measurement spots on the sample.

AFM measurements were performed using a Cypher ES system (Asylum Research, an Oxford Instruments Company) in intermittent-contact mode (AC mode) with photothermal excitation at 25 °C in air. SCOUT 70 RAu cantilevers (NuNano; ∼2 N/m spring constant, ∼70 kHz resonance frequency, ∼5 nm tip radius) were used. Images (512 × 512 pixels) were acquired at a scan rate of 1.95 Hz and 0° scan angle (i.e., fast scan axis was along the cantilever axis). Samples were glued on mounting pucks using 101RF replicating compound (Microset Products). For image analysis, data (z-sensor retrace) were processed in Gwyddion Free SPM analysis software version 2.60,^63^ including plane leveling, scar removal, row alignment (median of differences or polynomial) and zero adjustment. Root Mean Square (RMS) roughness values were extracted to characterize the topography of the respective regions. Thicknesses of scratched areas were obtained from 20-pixel-wide averaged line profiles. The 20-pixel-wide averaged line profiles were taken to determine the mean and standard deviation values for scratched and intact portions, respectively, and then their difference was determined.

## Results and Discussion

### Grafting PFcMA Brushes Via SI-ATRP

In this study, PFcMA polymer brushes were first grafted onto planar substrates using SI-ATRP, following the previously described method.^60,61^ However, the maximum thickness of the PFcMA brush reported from this method was only 46.3 ± 0.3 Å (4.6 nm).^61^ The initial goal of our research was to investigate whether the SI-ATRP conditions could produce thicker PFcMA brushes on the Si wafer substrate, as our final goal is to modify the ITO surface for future applications. A thicker PFcMA brush layer could enhance the electrochemical signal and enable broader applications by increasing the amount of ferrocene in the polymer chain. To achieve this, Si wafer substrates were treated with varying FcMA concentrations while maintaining a consistent ratio of scarified initiator, monomer, catalyst, and ligand (*t*-BbiB:FcMA:CuCl:PMDETA = 1:300:5:5) as described in the experimental section and Figure 1b. The results presented in Table 1 indicate that the molar mass *M*_n_ of the PFcMA free polymer increased with the FcMA concentration used in the reaction. The dispersity values (*Đ*) for all obtained free polymers were approximately 1.3, typical for a reversible deactivation radical polymerization technique like ATRP. The PFcMA1 sample synthesized from the lowest FcMA concentration reaction, under conditions similar to previous reports,^60,61^ yielded the thinnest brush thickness of 4.3 ± 0.6 nm, aligning with earlier studies.^61^ The polymer brush thickness rises significantly with higher monomer concentrations: 8.7 ± 0.5 nm (PFcMA2) and 8.9 ± 0.2 nm (PFcMA3) respectively. This trend can be attributed to an increased monomer concentration, resulting in longer polymer chains. When these longer polymer chains were grown from a surface (as in polymer brushes), they extended further from the surface, resulting in a thicker brush layer. Although *M*_n_ continued to increase from PFcMA2 to PFcMA3, the brush thickness showed no significant difference compared to the significant jump from PFcMA1 to PFcMA2. This observation suggests that at higher monomer concentrations, additional factors (such as grafting density, crowding effects or limitations of the surface-initiated reaction) might start to play a more significant role, potentially leading to a slight plateau or reduced efficiency in further increasing brush thickness, even though the individual polymer chains in solution continued to grow longer. Consequently, the condition with 0.6 M FcMA was selected to modify the ITO surface, achieving a PFcMA brush thickness of approximately 8.7 ± 0.5 nm (assuming the thickness remains consistent with that of a Si wafer substrate). The PFcMA brush-modified ITO, derived from this SI-ATRP method, is referred to as ITO-ATRP. This sample was used for further studies to compare its surface and electrochemical properties with the PFcMA brush-modified ITO obtained via Cu^0^-SI-ATRP.

**Table 1:** Summarized Molar Masses (*M*_n_) and Dispersity Values (*Đ*) of the Free Polymer (PFcMA) from SI-ATRP, and Polymer Brush Thickness on the Modified Si Wafer from Each Condition

Sample	FcMA conc. (*M*)^a^	*M*_n_ (g mol^–1^)^b^	*Đ* ^c^	Thickness (nm)^d^
PFcMA1	0.3	14,200	1.28	4.3 ± 0.6
PFcMA2	0.6	19,000	1.32	8.7 ± 0.5
PFcMA3	0.9	22,000	1.27	8.9 ± 0.2
ITO-ATRP	0.6	26,600	1.30	-

a Concentration of FcMA used in the SI-ATRP.

b Molar masses of free polymer (PFcMA) determined by SEC (PS standards, THF).

c Đ values determined by SEC in THF.

d Brush thickness measured by an ellipsometer.

### Model Study of Grafting PFcMA Brushes Via Cu^0^-SI-ATRP

In this study, polymer brushes (PFcMA) were first grafted onto Si wafer as model substrates using Cu^0^-SI-ATRP through three methods: the load method, the spacer method, and the filter paper method. The aim was to determine the most effective technique for polymerizing the FcMA monomer on a planar substrate before we turn our investigations to the conductive ITO substrates. Initially, the polymerization reactions were conducted as described by McGaughey et al.,^57^ involving the application of a reaction solution onto a copper plate, followed by placing the substrates between the spacer and the copper plate. However, this reaction setup provided an uneven surface on the PFcMA brush-modified substrates. The slow evaporation of the DMSO solvent caused FcMA to recrystallize during the reaction. Although DMSO has a very high boiling point (189 °C), the monomer concentration used in this study was high (close to its saturation point), and the reaction setup was not perfectly sealed. This resulted in the slow evaporation of DMSO over the 5 h reaction period from the small, exposed surface area at the edge of the substrate. This small loss of DMSO was enough to locally raise the monomer concentration to a supersaturated level and trigger the nucleation of crystals from this area. Areas with the remaining reaction solution exhibited faster polymerization and a thicker polymer brush layer, whereas the recrystallized regions showed little to no reaction, as illustrated in Figure S1. Therefore, the improved experimental setups have been created for the FcMA monomer to minimize solvent evaporation and prevent FcMA recrystallization. The configurations of the three methods are illustrated in Figure 1c. For these experimental setups, a 5 h reaction time was investigated. No recrystallization of FcMA occurred, and the polymer brush layer covered the entire surface of the modified substrate. Ellipsometry was used to measure the thickness of the polymer brush, revealing that the filter paper method resulted in the thickest polymer layer, followed by the spacer and load methods, respectively (Figure 2). The spacer method yields thicker brush thickness compared to the load-based method, consistent with previous studies involving other monomers, which indicated that brush thickness increases with distance *d* (*d* ≤ 0.5 mm) when the reaction occurs in a “sandwich-like” sealed setup, with a copper plate facing the initiator-functionalized substrate.^53,57,64^ This trend is due to the interplay of diffusion-controlled catalyst transport and reduced termination kinetics. In a confined reaction setup (*d* ≤ 0.5 mm), the diffusion of Cu­(I) activators from the copper plate to the substrate slows down as *d* increases. This results in lower local radical concentrations at larger distances, which in turn reduces the likelihood of termination reactions. Consequently, a more controlled and sustained polymerization process is facilitated, prolonging the lifespan of growing chains and leading to the formation of thicker brushes.^53,64^ However, with the filter paper-based method, although *d* was smaller than that in the case of the spacer method, the brush thickness achieved after the reaction was significantly larger than that obtained from the spacer method. These results align with previous studies that have shown filter paper accelerates brush growth by creating a confined reaction environment.^54^ It serves as both a reservoir for the monomer solution and a spacer, maintaining close proximity between the copper source and the substrate. This setup promotes controlled diffusion of Cu­(I) activators, limits oxygen interference, and reduces termination reactions, all of which support sustained radical polymerization. Consequently, this method yields much thicker polymer brushes in a shorter time frame.^54,57^

**Figure 2:**
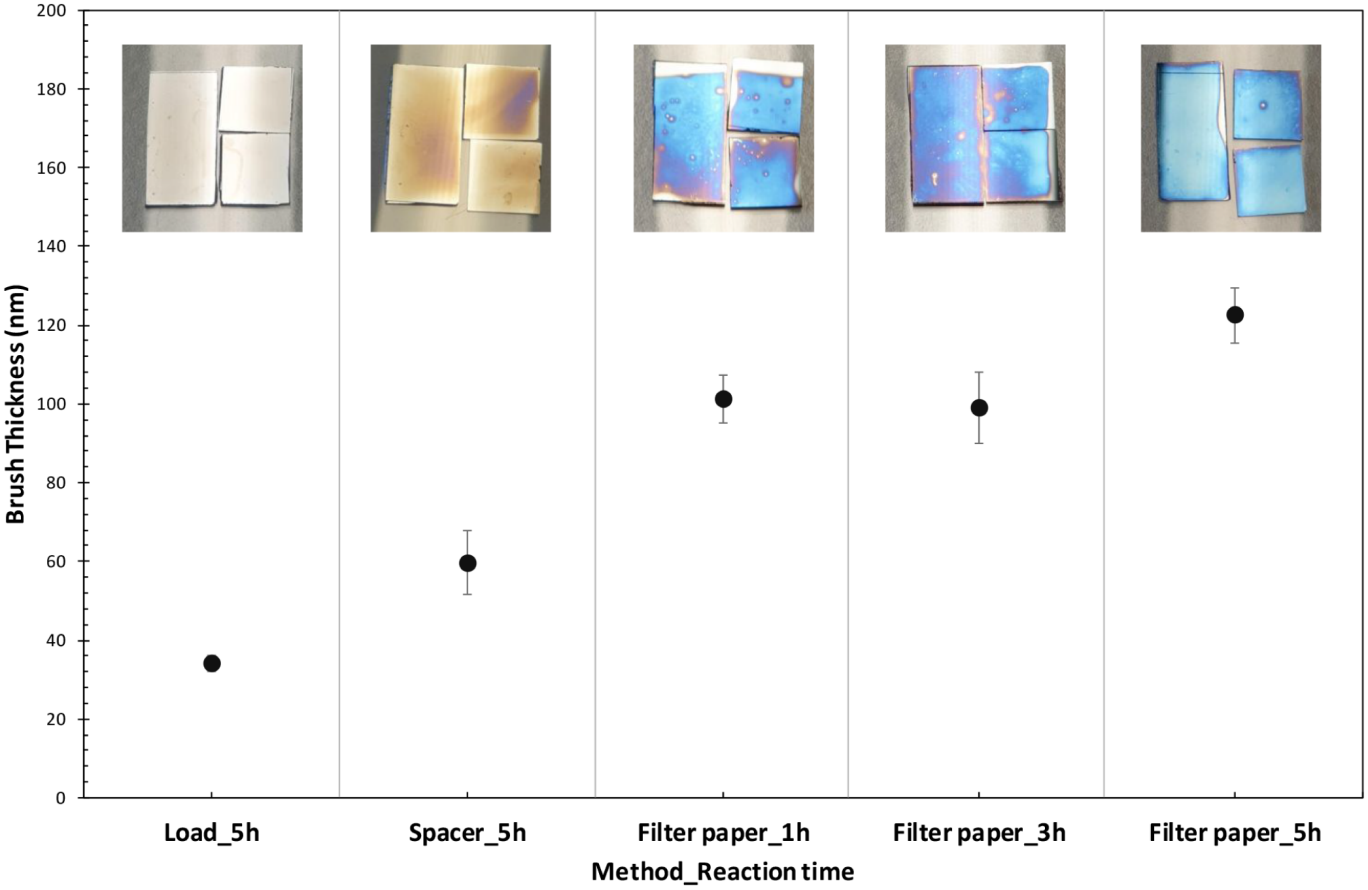
Brush thickness of PFcMA brush on the modified Si wafer surface, produced by Cu^0^-SI-ATRP through various methods: load, spacer, and filter paper methods. The brush thickness was measured by an ellipsometer, and the error bars represent SD. Insets illustrate optical images of the PFcMA-modified substrate obtained from each method.

In this study, we selected the filter paper method for further exploration because it produced the thickest polymer brush layer within the same reaction time. Therefore, the grafting of PFcMA polymer brushes using the filter paper method with varying reaction times of 1, 3, and 5 h was investigated. The findings indicate that a polymer brush thickness of 101 ± 6 nm can be achieved within 1 h, remaining consistent at this thickness for 3 h (99 ± 9 nm), and then slightly increasing to 122 ± 7 nm at 5 h. However, the inconsistent blue color tone and small, colorless spots on the surfaces of the modified substrates observed after 1 and 3 h suggest the inhomogeneity of the polymer brush layer (see Figure 2). Consequently, the 5 h reaction time was selected to ensure a uniform polymer brush layer for subsequent studies.

Figure 3a illustrates the ATR-FTIR spectra confirming the successful grafting of PFcMA onto Si wafer substrates through Cu^0^-SI-ATRP. The FTIR spectrum of the Si wafer reveals characteristic peaks associated with the thin SiO_2_ layers, including a peak at 610 cm^–1^ indicating deformations in the Si–O–Si network, a pronounced asymmetric Si–O–Si stretch at 1107 cm^–1^, and a symmetric Si–O–Si stretch near 893 cm^–1^. The FTIR spectra of both the Si wafer and SW-I displayed no significant differences. This similarity may be due to the subtle characteristic peaks of the amide group from the initiator being concealed by the Si wafer IR peaks in ATR mode, which is attributable to the very thin initiator layer of about 3 nm, as measured by an ellipsometer. All PFcMA-modified substrates derived from Cu^0^-SI-ATRP through various methods exhibited strong FTIR characteristic peaks of PFcMA (Figure S2). This includes the ester C=O stretch at 1718 cm^–1^ and C–O stretches at 1277 cm^–1^, indicative of methacrylate ester functionality, along with CH_2_ scissoring or C=C deformation at 1458 cm^–1^ associated with polymer backbone or Cp ring deformation. These observations confirmed the successful grafting of PFcMA brushes onto the Si wafer. The peak intensities were highest for the modified substrate obtained via the filter paper method, followed by those from the spacer and load methods, respectively. Additionally, PFcMA-modified substrates produced using the filter paper method at varying reaction times also displayed these characteristic peaks, with no significant differences in intensity. Thus, this aligns with the brush thickness results: the greater the thickness of the PFcMA brushes, the higher the intensity of the FTIR peaks linked to PFcMA functionality.

**Figure 3:**
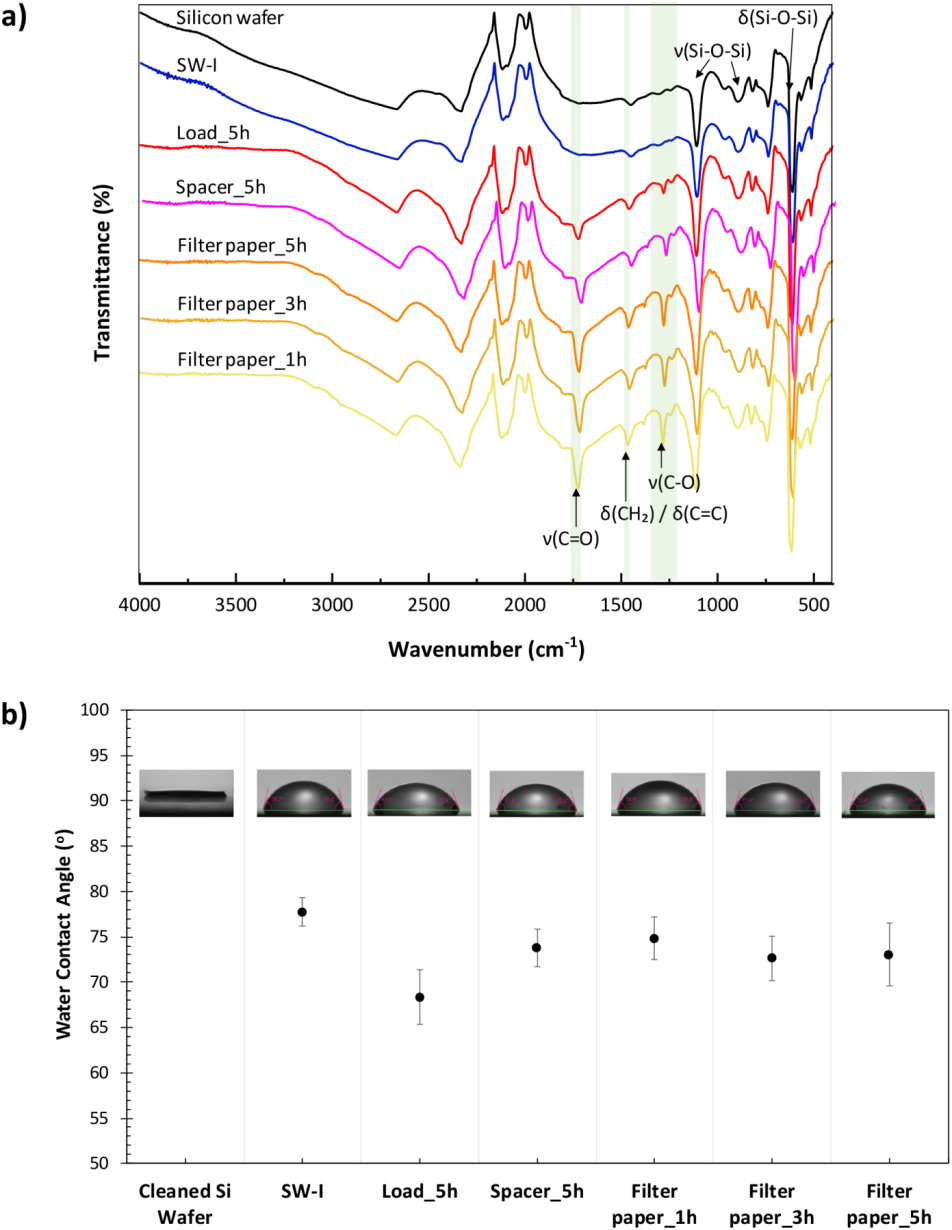
ATR-FTIR spectra (a) and WCA (b) for the Si wafer, the ATRP initiator grafted Si wafer (SW-I), and PFcMA brush-modified Si wafers by Cu^0^-SI-ATRP across various methods: load, spacer, and filter paper (5 h reaction time), along with PFcMA brush-modified Si wafers using the filter paper method with 1 and 3 h reaction times.

WCA of the cleaned Si wafer, SW-I, and the PFcMA brush-modified Si wafer by Cu^0^-SI-ATRP were also investigated (Figure 3b). The cleaned Si wafer exhibited a very low WCA (0–10°) due to its highly hydrophilic surface characterized by a native oxide layer and numerous surface hydroxyl groups (−OH).^65^ Following the ATRP initiator grafting steps, the resulting SW-I displayed increased hydrophobicity, as evidenced by a higher WCA (78° ± 2°) compared to that of the cleaned Si wafer, attributable to the silanized surface featuring the hydrophobic group of the initiator molecule. The subsequent grafting of the PFcMA brushes onto SW-I via Cu^0^-SI-ATRP resulted in a decrease in WCA, making the surface less hydrophobic than SW-I, regardless of the method employed (load, spacer, or filter paper). Although ferrocene itself is nonpolar,^66^ the overall polymer structure and its grafting behavior on the surface will determine the surface’s wettability. The observed reduction in WCA after grafting could be due to the presence of ester groups within the polymer side chains of PFcMA. When the polymer with hydrophilic ester groups was grafted onto SW-I, it effectively increased the surface polarity, thereby enhancing its wettability by water. The load method yielded the lowest contact angle and was the most hydrophilic among the grafted samples compared to the other methods. Other modified samples obtained through spacer and filter paper methods exhibited no significant difference in contact angles (∼73–75°) but demonstrated lower hydrophilicity than the sample obtained via the load method. The differences in WCA reflect variations in grafting efficiency, polymer density, and/or polymer conformation achieved by each method, leading to different degrees of exposed ester groups and consequently varying levels of hydrophilicity.

### Grafting PFcMA Brushes Via Filter Paper-Assisted Cu^0^-SI-ATRP

In the next step and for further improvement of the PFcMA-immobilization strategy, polymer brushes (PFcMA) were grafted onto silica wafer substrates using the Cu^0^-SI-ATRP with filter paper-based method (FP-Cu^0^-SI-ATRP). The effect of monomer concentration on the properties of PFcMA brush-modified Si wafers was investigated. Therefore, the reactions were employed using different FcMA concentrations of 0.07 M, 0.15 M, 0.3 M, and 0.6 M, with 0.0012 M PMDETA in DMSO for 5 h. In these reactions, cellulose filter paper with a thickness of 0.16 mm was used as a spacer between the Cu plate and SW-I, as described in the experimental section. The resulting PFcMA-modified Si wafers were labeled as FP_0.07M, FP_0.15M, FP_0.3M, and FP_0.6M, respectively. As shown in Figure 4a, the thickness of the PFcMA brush increased linearly with an increase of the FcMA concentration. This can be attributed to the elevated monomer concentration, which accelerates the propagation step, facilitating faster chain growth and resulting in thicker brushes.^47^ In grafting from polymerization, the initiator grafting density determines the number of available sites for chain growth. Therefore, polymer grafting density is directly proportional to initiator grafting density. The Cu^0^-SI-ATRP method is known for its ability to maintain a high concentration of active polymer chain ends. As a result, chain termination is minimized, leading to nearly every surface-bound initiator growing a polymer chain. This results in a polymer grafting density that is almost equal to the initiator grafting density. In this work, we assumed that all prepared initiator-functionalized substrates had the same amount of initiator grafting density because they were prepared using the same method and conditions. Consequently, the observed linear increase in brush thickness was a direct result of the higher monomer concentration, which allowed the growth of longer polymer chains with higher molecular weight (*M*_n_). These longer chains required a more extended conformation to accommodate their larger size at a constant grafting density, resulting in a thicker brush. Thus, under this polymerization method and the corresponding reaction conditions, the thickness of the desired PFcMA brush can be regulated by adjusting the concentration of the FcMA monomer. The polymer brush layer on the surface displayed different colors due to variations in brush thickness, as shown in Figure 4d. All modified samples exhibited a uniform surface color, indicating good coverage of the PFcMA brush layer on the substrate surface. The WCA (Figure 4b) showed no significant difference. The ATR-FTIR spectra (Figure 4c) of the four modified substrates displayed characteristic peaks associated with PFcMA. As expected, the peak intensity increased with the FcMA concentration in the polymerization reaction, due to the increase in brush thickness.

**Figure 4:**
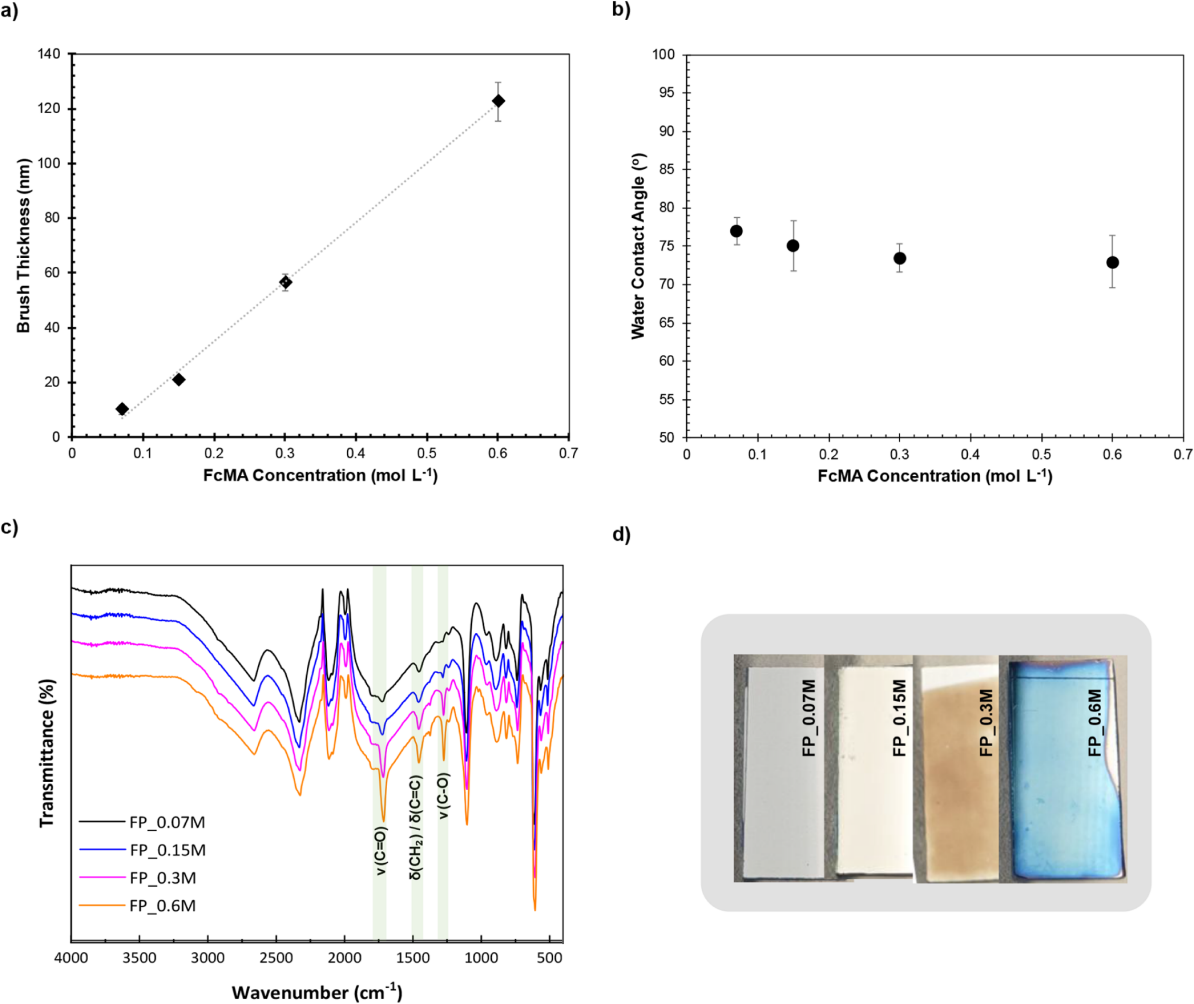
(a) PFcMA brush thickness measured by an ellipsometer (*R* ^2^ = 0.9973), (b) WCA (error bars represent SD), (c) ATR-FTIR spectra, and (d) photographs of the PFcMA-modified Si wafers obtained via FP-Cu^0^-SI-ATRP with varying FcMA monomer concentrations.

The findings indicate that the PFcMA brush, grafted from a flat substrate, can be synthesized through FP-Cu^0^-SI-ATRP. This process achieves brush thicknesses, measured by ellipsometer, ranging from 10 to 122 nm by adjusting the concentration of the FcMA monomer in the reaction. For further investigation, this technique was employed to modify ITO-coated glass substrates, resulting in PFcMA brush-modified ITO with varying brush thicknesses, whose electrochemical properties were analyzed. Additionally, PFcMA brush-modified ITO obtained via the conventional SI-ATRP method (ITO-ATRP) was used for comparison purposes.

### PFcMA Brush-Modified ITO Substrates

Herein, the PFcMA brush-modified ITO samples were produced by the FP-Cu^0^-SI-ATRP using different FcMA concentrations as described previously, resulting in four modified ITO samples with different brush thicknesses: 10.1 ± 1.7 nm (ITO-FP_0.07M), 21.1 ± 0.9 nm (ITO-FP_0.15M), 56.4 ± 3.1 nm (ITO-FP_0.3M), and 122.6 ± 7.1 nm (ITO-FP_0.6M). Moreover, one modified ITO sample, prepared using SI-ATRP (ITO-ATRP, with a brush thickness of 8.7 ± 0.5 nm), was also investigated for comparison purposes. The brush thickness for all these PFcMA-modified ITO samples was assumed to be the same as that of the PFcMA-modified Si wafers obtained from the same polymerization method.

The UV–vis data shown in Figure 5 reveal that the thickest PFcMA brush-modified ITO (ITO-FP_0.6M) exhibits a prominent absorption peak for ferrocene units at 455 nm. This observation aligns with the reference UV–vis spectrum for FcMA and PFcMA (Figure 5b), which represents the reduced form of ferrocene.^67^ Additionally, a shift in the ferrocene absorption peak to a shorter wavelength (a blue shift) was observed when the PFcMA brush thickness on ITO substrates decreased. This phenomenon can be attributed to the increased packing density of the ferrocene units (as the brush thickness decreases, the ferrocene units on the polymer side chains are likely forced into closer physical proximity), which enhances interferrocene electronic interactions. Such interactions often foster the development of H-aggregates, which occur when chromophores, such as ferrocene units, are arranged in a face-to-face manner, thereby facilitating strong excitonic coupling. Within H-aggregates, the transition dipole moments of neighboring chromophores align in a way that increases the energy of the excited state, leading to absorption at higher energies (shorter wavelengths) compared to isolated ferrocene units.^68,69^ Additionally, the rigid and confined environment of the thinner polymer layer (in the solid state) intensifies these close-range interactions, making them the key factor driving the observed spectral shift.

**Figure 5:**
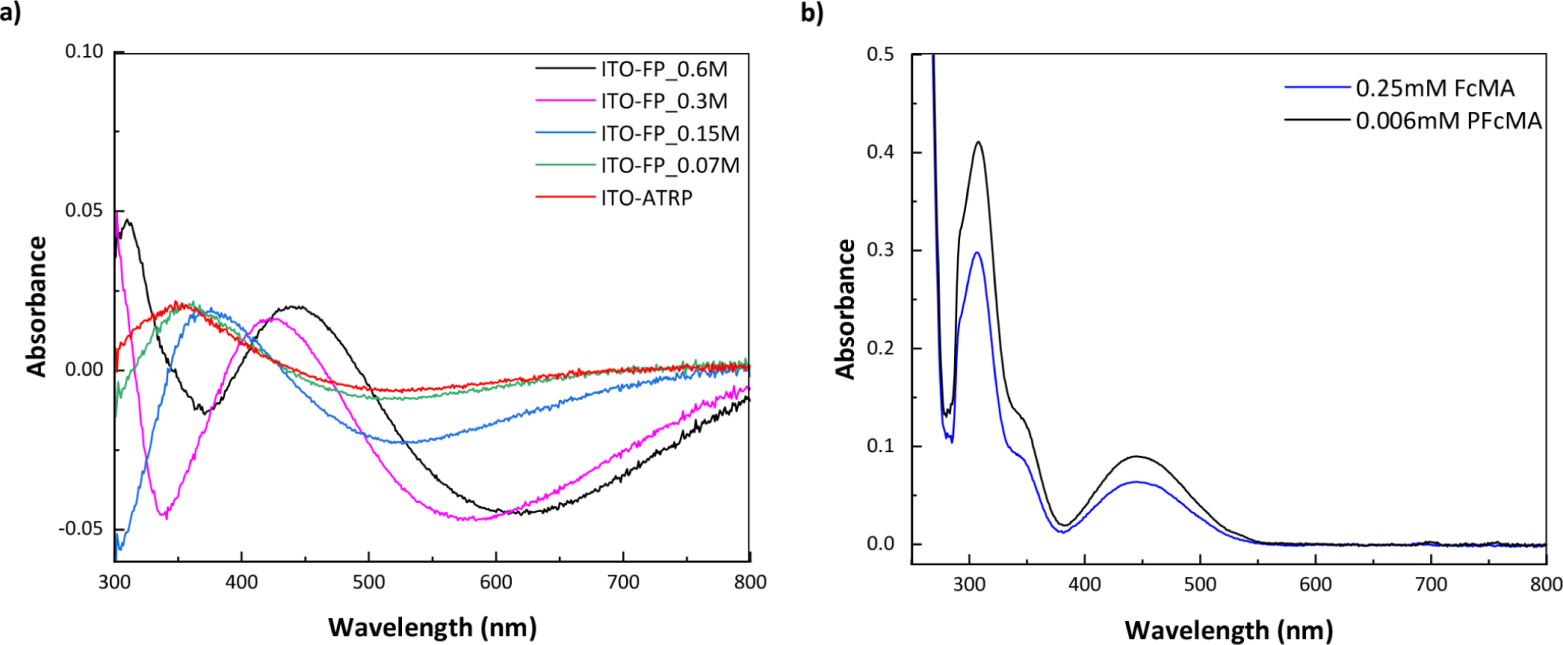
a) UV–vis spectrum of PFcMA brush-modified ITO. b) the reference UV–vis spectrum of FcMA and PFcMA (*M*_n_ = 19 000 g mol^–1^) in THF.

Based on the ATR-FTIR results (Figure 6a), the ITO-coated glass substrate displayed peaks characteristic of a standard ITO film, including a broad, strong peak of In–O and Sn–O stretching around 500–700 cm^–1^ and a broad peak around 1680 cm^–1^ of H–O–H bending due to ambient water. Following initiator grafting, the ITO-I still largely resembled pure ITO, with no significant changes noted, likely due to the very thin initiator layer, which was approximately 3 nm thick. The PFcMA brush-modified ITO samples obtained through FP-Cu^0^-SI-ATRP exhibited the characteristic IR peaks of PFcMA, including aliphatic C–H stretching at 2924 cm^–1^, the ester C=O stretch at 1724 cm^–1^, C–O stretch at 1280 cm^–1^, and C–O–C stretch at 1142 cm^–1^, indicating methacrylate ester functionality; CH_2_ scissoring or C=C deformation at 1463 cm^–1^ associated with the polymer backbone or Cp ring deformation; and out-of-plane Cp ring deformation at 774 cm^–1^. These observations confirm the successful polymerization of PFcMA brushes onto the ITO substrate via FP-Cu^0^-SI-ATRP. It is clearly seen that the intensity of the ν­(C=O) peak of PFcMA at 1724 cm^–1^ gradually increased compared to the intensity of the ITO peak at 653 cm^–1^ as the FcMA concentration increased (from ITO-FP_0.07 M to ITO-FP_0.6M), which suggests a relatively higher amount of PFcMA on the surface, as expected with increasing polymer brush thickness.

**Figure 6:**
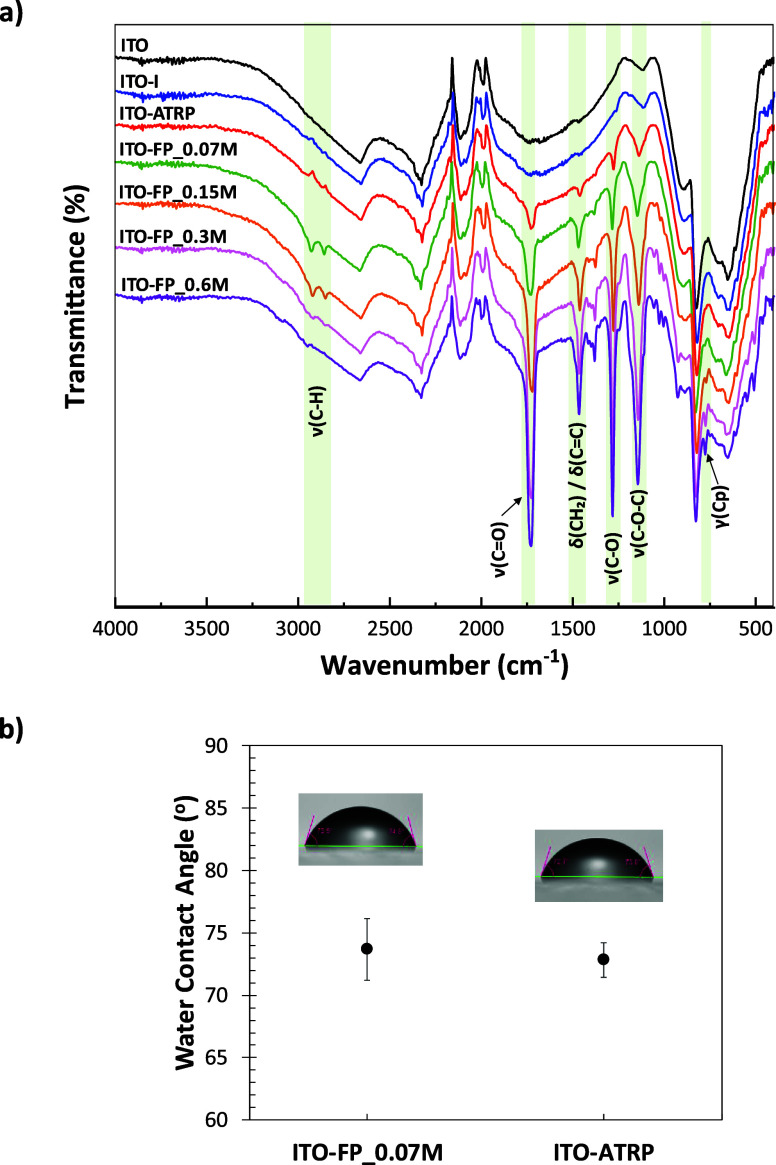
a) ATR-FTIR spectra of ITO, ITO-I, and PFcMA brush-modified ITO samples, b) WCAs of the modified ITO prepared from FP-Cu^0^-SI-ATRP (ITO-FP_0.07M) and from SI-ATRP (ITO-ATRP) with comparable thickness.

The WCA of the modified ITO samples, obtained via the filter paper method, was examined. The results indicate no significant difference between grafting from Si wafer and ITO substrates under identical conditions (Figure S3). When comparing ITO produced using two different methods, FP-Cu^0^-SI-ATRP (ITO-FP_0.07M) and SI-ATRP (ITO-ATRP), the ATR-FTIR spectrum of ITO-ATRP exhibited the same characteristic peaks of PFcMA as ITO-FP_0.07M, albeit with lower peak intensities (Figure 6a). This reduction may be attributed to the slightly thinner brush thickness of ITO-ATRP (8.7 ± 0.5 nm) compared to ITO-FP_0.07 M (10.1 ± 1.7 nm). Additionally, there were no significant difference in WCA between these two samples (Figure 6b).

The atomic force microscopy (AFM) images (Figure 7) reveal significant differences in surface morphology and roughness across the various ITO substrates. The pristine ITO substrate exhibited a root-mean-square (RMS) roughness of 2.9 nm, and after introducing an initiator layer (ITO-I), the RMS roughness remained the same (3.0 nm). Subsequent SI-ATRP polymerization (ITO-ATRP) further decreased RMS roughness to 2.6 nm, indicating a relatively smooth polymer brush formation with a thickness of 6.2 ± 1.7 nm, measured by AFM (Figure S4). The polymerization using the FP-Cu^0^-SI-ATRP at increasing concentrations of FcMA (ITO-FP_0.07M, ITO-FP_0.15M, ITO-FP_0.3M, ITO-FP_0.6M) generally led to a reduction in RMS roughness compared to pristine ITO, reaching a minimum of 1.8 nm for ITO-FP_0.15M. Furthermore, the characteristic grain structure of the ITO substrate is increasingly covered by the PFcMA brushes as the concentration of FcMA increases. Concurrently, the brush thickness systematically increases with higher FcMA concentrations (Figures 7 and S4), ranging from 4.2 ± 2.9 nm for ITO-FP_0.07 M to 81.0 ± 7.9 nm for ITO-FP_0.6M, suggesting the successful and tunable grafting of PFcMA brushes onto the ITO surface. The thickness values obtained from AFM measurements correlate well with those obtained from ellipsometry on PFcMA-modified Si wafers prepared using the same polymerization method and conditions.

**Figure 7:**
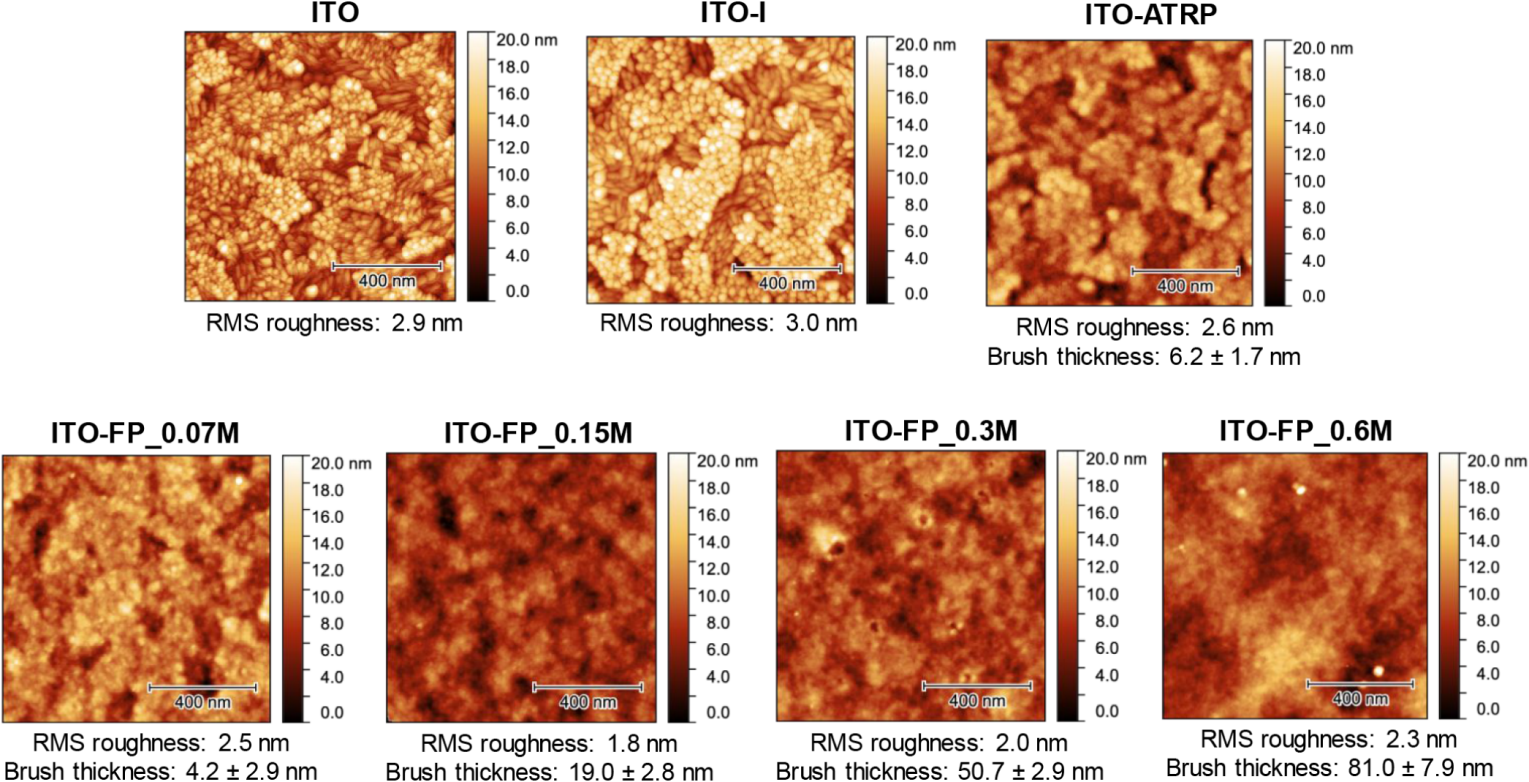
AFM images and corresponding root-mean-square (RMS) roughness values of pristine ITO, ITO with initiator (ITO-I), and PFcMA brush-modified ITO samples. All images have a scale bar of 400 nm. The RMS roughness and brush thickness (obtained from the AFM topography images of scratched samples, as given in Figure S4) are indicated for each substrate.

The electrochemical properties of all PFcMA brush-modified ITO samples were examined, including those prepared through FP-Cu^0^-SI-ATRP: ITO-FP_0.07M, ITO-FP_0.15M, ITO-FP_0.3M, and ITO-FP_0.6M, as well as samples made via the SI-ATRP method: ITO-ATRP. Electrochemical switching is expected to change the polymer conformation. However, this effect is strongly correlated with the solvent and the counterion type. Acetonitrile, a standard solvent for measuring ferrocene in electrochemistry, was used for CV measurements. In this solvent, the polymer brush is expected to exhibit only minor conformational changes upon oxidation, allowing us to focus on the electrochemical property study of the PFcMA brushes. Although osmotic pressure changes are expected upon charging, we observed no loss of polymer chains from the substrates. Cyclic voltammograms (Figure 8a) and Randles-Sevcik plots (Figure 8b) illustrate the electrochemical characteristics of all five ITO-modified samples. Figure 8a shows that the peak current magnitudes for the ITO-FP series (0.07 M to 0.60 M) directly correlate with increasing monomer concentration and polymer brush thickness. The peak currents reached approximately 1.5 mA (anodic) and −1.5 mA (cathodic) for the sample with the thickest PFcMA layer (ITO-FP_0.6M, 122.6 ± 7.1 nm). In contrast, the sample with the thinnest PFcMA layer (ITO-FP_0.07M, 10.1 ± 1.7 nm) had significantly lower peak currents, around 0.3 mA (anodic) and −0.3 mA (cathodic). Thus, ITO-FP_0.6M exhibited the highest electrochemical activity, producing the largest currents, consistent with expectations for thicker layers of electroactive material. However, the relationship between peak current (*i*_p_) and brush thickness (Figure S5) demonstrated a nonlinear increase in peak current with increasing brush thickness. For both oxidation and reduction, the current initially rose relatively steeply as thickness increased from approximately 10 to 60 nm. However, as the thickness continued to increase beyond 60 nm, the rate of current increase significantly diminished, suggesting a saturation or leveling-off effect at higher film thicknesses. This behavior is characteristic of systems where charge transport within the film becomes the rate-limiting step. While a thicker film contains more electroactive material, the ability of charge (electrons and/or counterions) to move efficiently through the entire thickness to participate in the redox reaction becomes progressively hindered. This often indicates a transition from a regime where all sites are easily accessible to one where diffusion or migration within the bulk of the film restricts the overall current, rather than a direct proportionality that would be observed in purely surface-confined or very thin films.

**Figure 8:**
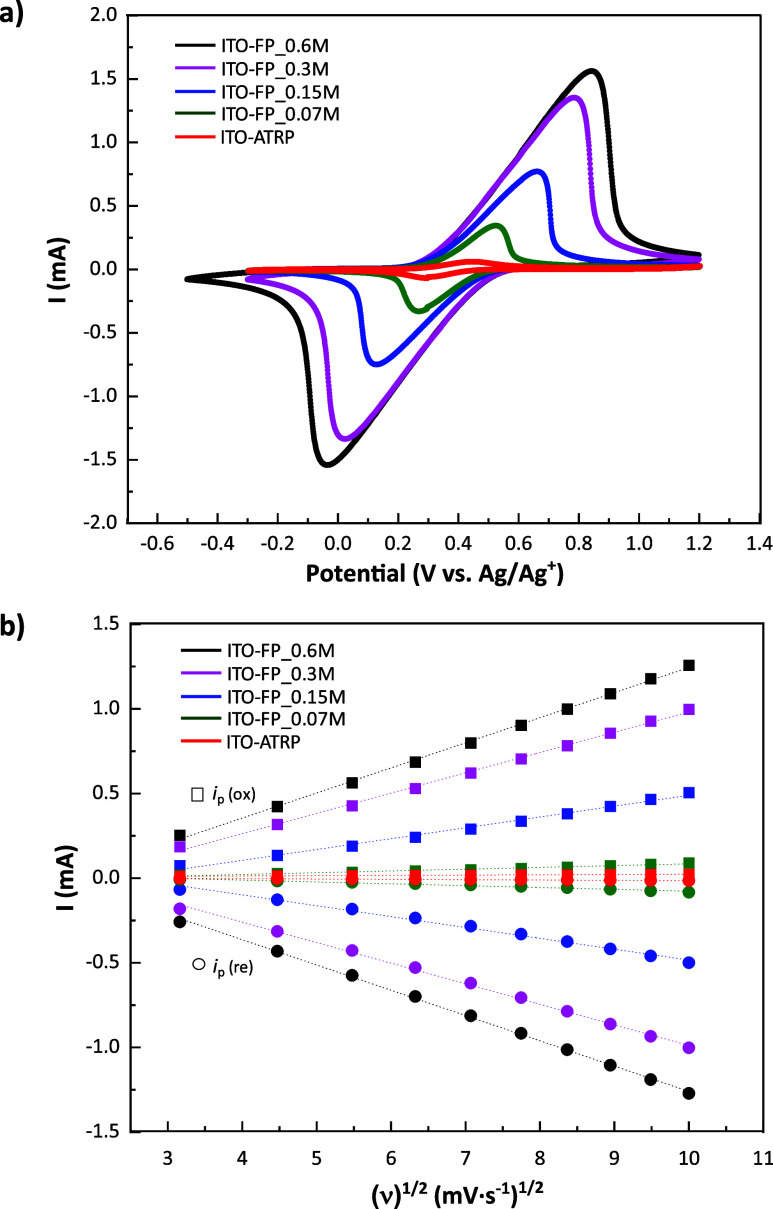
a) Cyclic voltammograms of PFcMA brush-modified ITO samples in acetonitrile with a [TBA]­[PF_6_] electrolyte, with Ag/Ag^+^ reference and Pt counter electrodes, at a scan rate of 100 mV·s^–1^. b) Randles–Sevcik plot of PFcMA brush-modified ITO samples.

In examining peak potential separation (Δ*E*_p_) and shape, ITO-FP_0.07 M displayed relatively sharp peaks with minimal separation, suggesting a more electrochemically reversible or quasi-reversible process. As the thickness of the brush increased, the peaks became broader and more asymmetric, with a noticeable rise in peak separation, especially in ITO-FP_0.6M, which exhibited the largest Δ*E*_p_ (∼0.87 V). This suggests that while increased material loading boosts current, it also brings substantial challenges from ohmic resistance (*iR* drop) and/or slower charge transport kinetics in thicker films. Notably, despite the peak broadening and increased separation observed in the thicker PFcMA brush layer ITO-FP samples, the linear nature of all Randles–Sevcik plots in Figure 8b is a crucial finding. This linearity indicates that the electrochemical processes for all five PFcMA-modified ITO are mainly diffusion-controlled within the examined scan rate range.^70^ Consequently, the rate-limiting step remains the transport of charge through the polymer film via electron hopping and ion migration, even as kinetic or resistive effects become more pronounced. The slopes of the linear fits are directly linked to the peak current magnitudes shown in Figure 8a. ITO-FP_0.6 M had the steepest slopes during both oxidation and reduction, followed by ITO-FP_0.3M, ITO-FP_0.15M, and ITO-FP_0.07M, respectively. The increase in slope for higher FcMA concentrations likely indicates a larger amount of active material or an enhanced apparent diffusion coefficient, possibly due to improved connectivity in slightly thicker films up to a certain threshold. Conversely, the ITO-ATRP sample showed significantly lower currents and broader peaks compared to ITO-FP_0.07 M (with similar brush thickness), and it exhibited lower slopes in its Randles–Sevcik plot, implying significantly less electroactive material or substantially poorer charge transport efficiency relative to the ITO-FP series. Although its CV shape may seem less distorted than that of the thickest polymer layer FP sample, its overall electrochemical activity was markedly lower.

To comprehensively understand the electrochemical behavior of ITO electrodes modified with PFcMA polymer brushes, CV experiments at various scan rates (10 mV·s^–1^ to 100 mV·s^–1^) were conducted (Figure 9). A clear trend emerged within the ITO-FP series. As the monomer concentration or brush thickness increased, the peak current magnitudes significantly rose, indicating a greater amount of electroactive material or a higher density of redox sites within the polymer brush layer. Concurrently, increasing concentration (and thus inferred film thickness) led to broader and more asymmetric peaks, along with a notable increase in peak potential separation (Δ*E*_p_). This suggests that while more material is present, kinetic limitations, slow charge transport within the thicker films, and/or increased ohmic resistance (iR_drop_) become more prominent, affecting the reversibility of the electrochemical process.

**Figure 9:**
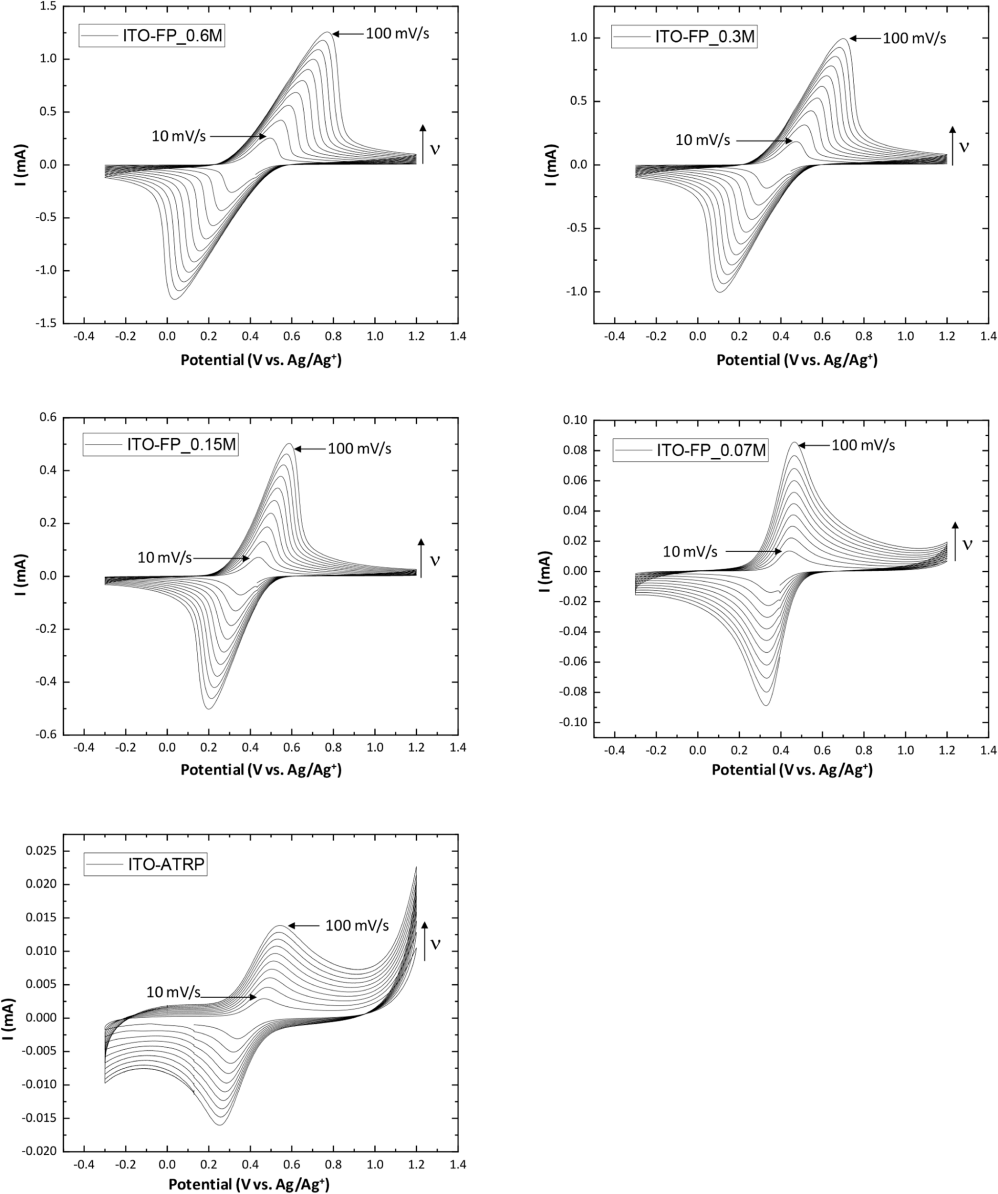
Experimental cyclic voltammograms at different scan rates (10 to 100 mV·s^–1^) for PFcMA brush-modified ITO samples in acetonitrile with a [TBA]­[PF_6_] electrolyte, with Ag/Ag^+^ reference and Pt counter electrodes.

Comparing ITO-FP_0.07 M prepared from FP-Cu^0^-SI-ATRP and ITO-ATRP prepared from SI-ATRP, which possess comparable brush thicknesses (10.1 ± 1.7 nm and 8.7 ± 0.5 nm, respectively), distinct differences in their electrochemical responses were observed. Although both exhibited an increase in peak current with scan rate, the ITO-FP_0.07 M sample showed significantly higher peak currents than ITO-ATRP. Furthermore, ITO-FP_0.07 M displayed sharper peaks with a smaller and more stable Δ*E*_p_, indicative of a more electrochemically reversible and efficient charge transfer process. In contrast, ITO-ATRP exhibited broader peaks and a larger, more scan-rate-dependent Δ*E*_p_, indicating slower electron transfer kinetics, higher internal resistance, or less efficient charge transport, despite its similar thickness. This comparison reveals that even at comparable film thicknesses, the specific polymer synthesis method (FP-Cu^0^-SI-ATRP vs SI-ATRP) has a profound impact on the polymer brush morphology, charge transport pathways, and interfacial properties, ultimately affecting its electrochemical performance.

In summary, the results collectively indicate that the method of polymer brush deposition has a significant impact on both the quantity of electroactive material and the efficiency of charge transport within the polymer film. The FP-Cu^0^-SI-ATRP appeared to yield PFcMA brushes with a higher loading of active material, and, particularly at a thin PFcMA brush layer (∼10 nm), very efficient charge transport kinetics, resembling those of ideal diffusion-controlled systems. However, increasing the FcMA monomer concentration in the FP-Cu^0^-SI-ATRP led to thicker films where resistive losses and charge transport limitations became pronounced, distorting the CVs despite the overall process remaining diffusion-controlled.

In contrast, the SI-ATRP method, even for a comparably thin FcMA brush thickness, produced a polymer brush layer with lower electrochemical activity and greater kinetic/resistive limitations. This suggests that the polymer brush architecture or polymer brush layer morphology created by SI-ATRP may inherently be denser, less permeable to ions, or have slower electron hopping pathways, thereby hindering charge transport more effectively than the FP-derived films. The FP-Cu^0^-SI-ATRP, particularly at the lower monomer concentration used, seems to strike a better balance between material loading and efficient charge transport for the specific redox system under study.

## Conclusion

The filter paper-assisted Cu^0^-SI-ATRP (FP-Cu^0^-SI-ATRP) provides a rapid, efficient, and tunable approach for grafting ferrocene-containing polymer brushes, such as PFcMA brushes, from planar substrates. The PFcMA brush thickness can be precisely controlled via monomer concentration, yielding uniform coatings ranging from 10 to 122 nm. Compared to other Cu^0^-SI-ATRP approaches, such as the load and the spacer methods, FP-Cu^0^-SI-ATRP offers superior polymerization efficiency, generating thicker brush layers within the same reaction time. In contrast to conventional SI-ATRP, FP-Cu^0^-SI-ATRP significantly reduces reagent consumption and reaction time from 20 h in SI-ATRP to 5 h in FP-Cu^0^-SI-ATRP, while eliminating the need for metal catalyst removal and achieving substantially greater film thickness.

The electrochemical performance of the obtained PFcMA brush-modified ITO exhibited a strong dependence on the thickness of the PFcMA brush. A thin brush layer (∼10 nm) prepared via FP-Cu^0^-SI-ATRP exhibited efficient charge transport and well-defined diffusion-controlled redox behavior. As the brush thickness increased, resistive limitations became more pronounced, though redox activity remained diffusion-dominated. Notably, ITO modified via SI-ATRP exhibited significantly lower electrochemical activity and inferior electron transfer kinetics, even at similar thicknesses, suggesting that the polymer brush architecture and permeability are critical determinants of electrochemical performance.

Therefore, these results highlight the FP-Cu^0^-SI-ATRP as a highly advantageous approach for engineering redox-active polymer interfaces with tunable electrochemical properties, making it promising for integration in a variety of advanced applications. Potential uses include smart electrochemical systems such as switchable chemical and biosensors, controlled-release surfaces, advanced separation and filtration via electro-sorption, electrochemical actuators, smart coating technology, and tunable electron-transfer interfaces.

## Supplementary Material



## Notes

EThe Supporting Information is available free of charge at https://pubs.acs.org/doi/10.1021/acsapm.5c02861.

Experimental setup and thickness results in the preliminary study of Cu^0^-SI-ATRP method, ATR-FTIR spectrum of PFcMA, WCA of PFcMA brush-modified ITO, AFM images for brush thickness measurements, and plots of peak current (*i*_p_) vs brush thickness (PDF)
