# Supplementary material for: Electrochemical Switching of Metallopolymer-Functionalized Indium Tin Oxide Derived by Cu 0 ‑Mediated Atom Transfer Radical Polymerization
Source: ACS Appl Polym Mater. 2025 Sep 9;7(18):12831–45. doi: 10.1021/acsapm.5c02861 (PMC12481626; doi:10.1021/acsapm.5c02861)
Supplement: Supplementary file 1 [file ap5c02861_si_001.pdf]

Supporting Information for

Electrochemical Switching of Metallopolymer-Functionalized Indium Tin Oxide Derived by Cu^0-Mediated Atom Transfer Radical Polymerization

Jaeshin Kim[1], Bizan N. Balzer[2,3], Markus Gallei[1,4], and Suteera Witayakran[1,5*]*

[1] Polymer Chemistry, Saarland University, Campus C4 2, 66123 Saarbrücken, Germany

[2] Institute of Physical Chemistry, University of Freiburg, Albertstr. 21, 79104 Freiburg, Germany

[3] Freiburg Materials Research Center (FMF), University of Freiburg, Stefan-Meier-Str. 21, 79104 Freiburg, Germany

[4] Saarene, Saarland Center for Energy Materials and Sustainability, Saarland University, 66123 Saarbrücken, Germany

[5] Max Planck Institute for Informatics, Saarland Informatics Campus, Building E1 4, 66123 Saarbrücken, Germany

Corresponding Authors:

*Markus Gallei – markus.gallei@uni-saarland.de

*Suteera Witayakran – suteera.witayakran@uni-saarland.de

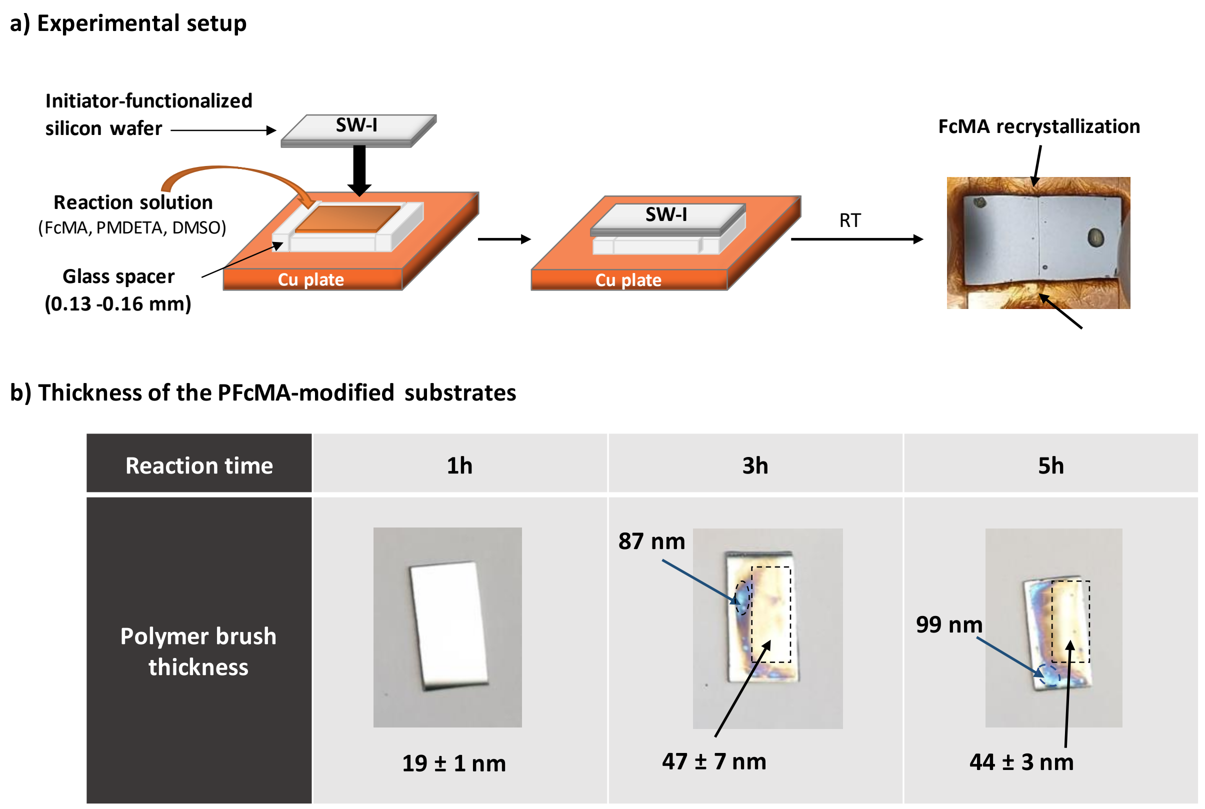

Figure S1. a) Schematic depiction of the experimental setup for Cu^0-SI-ATRP as detailed by McGaughey et al.,[1] with the image on the right illustrating the recrystallization of FcMA during the reaction. b) Images showcasing the PFcMA-modified substrates at various reaction times along with the polymer brush thickness.

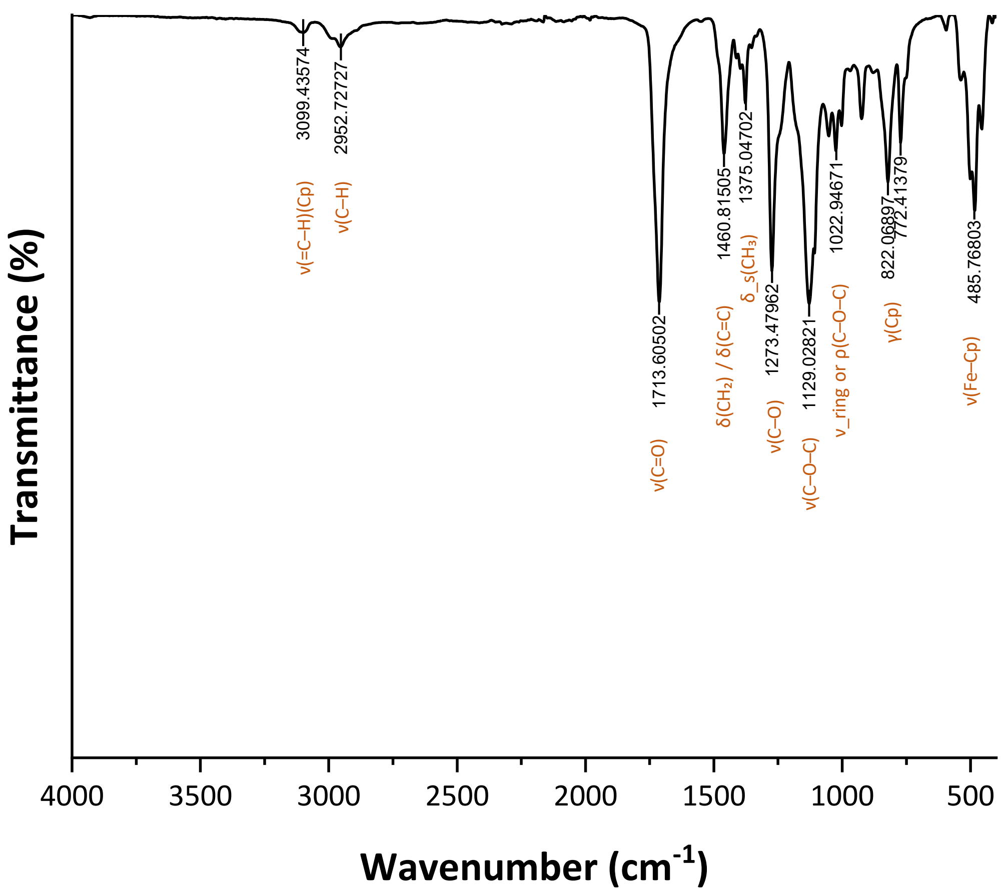

Figure S2. ATR-FTIR spectrum of PFcMA.

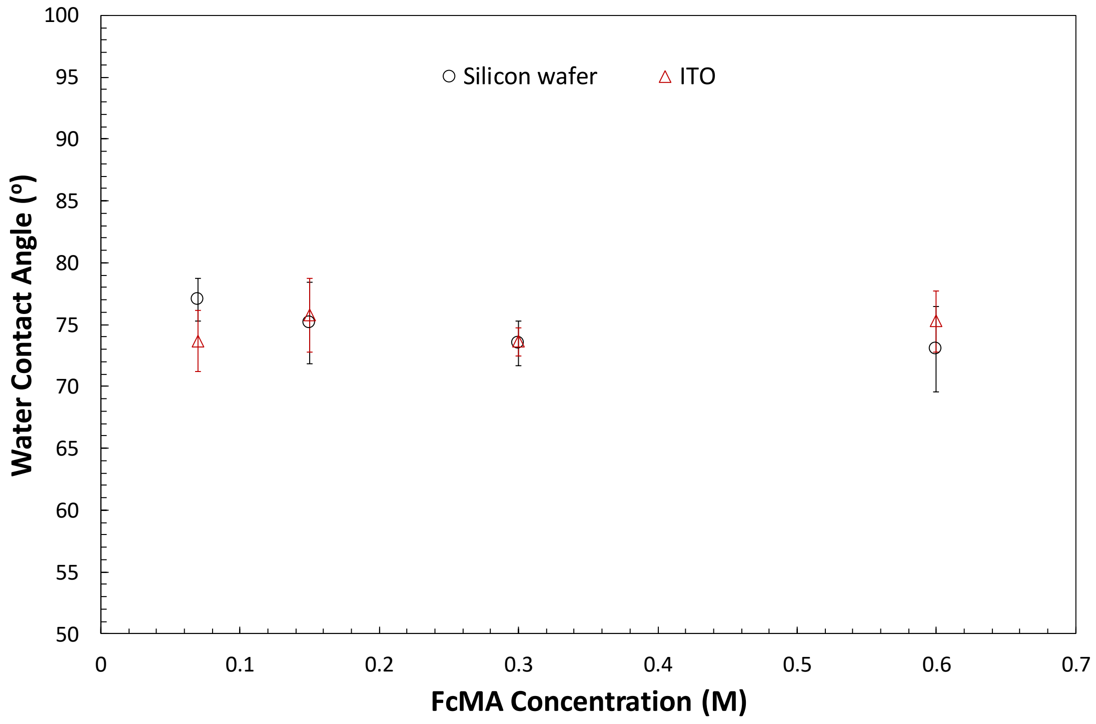

Figure S3. WCA of PFcMA brush-modified substrates prepared via FP-Cu^0-SI-ATRP using two different substrates: Si wafer and ITO-coated glass.

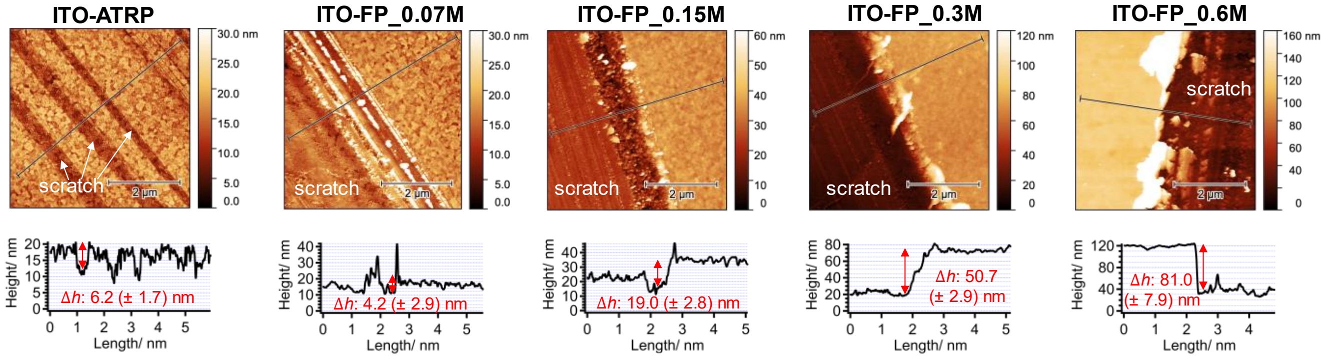

Figure S4. AFM topography images of the scratched PFcMA brush layer from the PFcMA brush-modified ITO samples and corresponding line profile across the scratched region used to measure brush thickness (Δh).

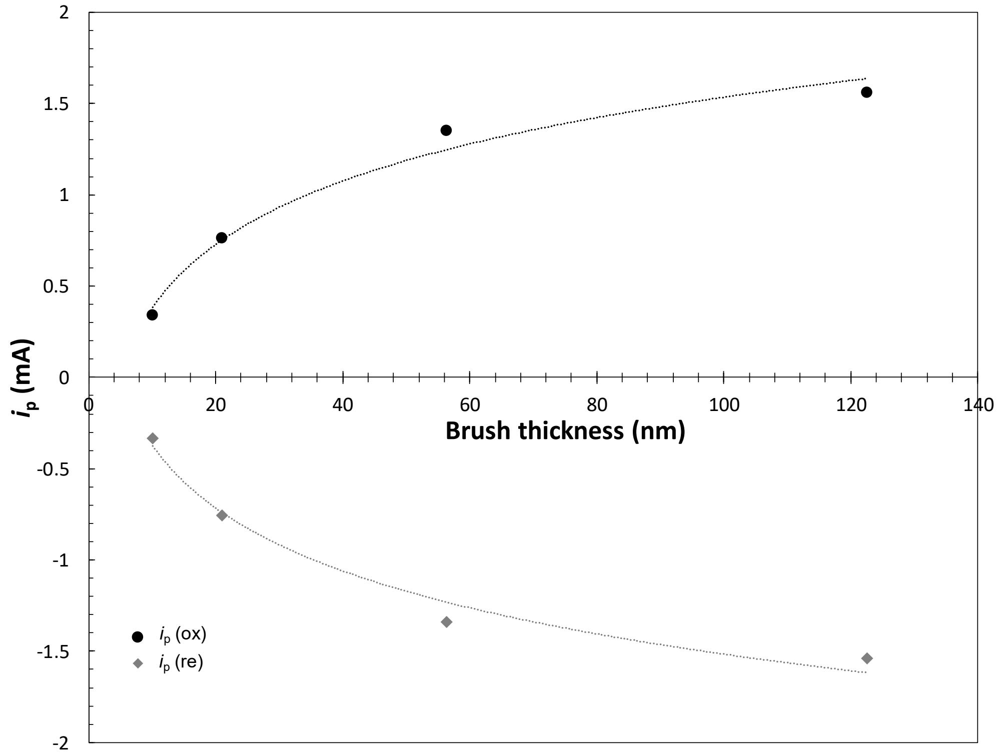

Figure S5. Plots of peak current (i_p) vs. polymer brush thickness of PFcMA brush-modified ITO samples prepared via FP-Cu^0-SI-ATRP.

References

1. McGaughey, A. L.; Srinivasan, S.; Zhao, T.; Christie, K. S. S.; Ren, Z. J.; Priestley, R. D. Scalable Zwitterionic Polymer Brushes for Antifouling Membranes Via Cu0-Mediated Atom Transfer Radical Polymerization. *ACS Appl. Polym. Mater.* **2023,** *5* (7), 4921-4932, DOI: 10.1021/acsapm.3c00407.
